# Low-dose Amiodarone Is Safe: A Systematic Review and Meta-analysis

**DOI:** 10.19102/icrm.2020.110403

**Published:** 2020-04-15

**Authors:** Ronpichai Chokesuwattanaskul, Nupur Shah, Susama Chokesuwattanaskul, Zhigang Liu, Ranjan Thakur

**Affiliations:** ^1^Sparrow Hospital, Michigan State University, Lansing, MI, USA; ^2^Faculty of Medicine, King Chulalongkorn Memorial Hospital, Chulalongkorn University, Bangkok, Thailand; ^3^St. Mary Mercy Hospital, Livonia, MI, USA; ^4^Department of Ophthalmology, Faculty of Medicine, Chiang Mai University, Chiang Mai, Thailand; ^5^Division of Cardiac Electrophysiology, University of Michigan Health Care, Ann Arbor, MI, USA

**Keywords:** Amiodarone, low dose, meta-analysis, safety, side effect

## Abstract

Amiodarone is commonly used for a variety of arrhythmias and, in some parts of the world, is the only available antiarrhythmic drug (AAD). Yet, amiodarone is known to have a wide range of potential side effects, many of which are dose- and duration-dependent. We sought to study the incidence of side effects leading to the discontinuation of low-dose amiodarone, arbitrarily defined as 200 mg/day or less, and very-low-dose amiodarone, defined as 100 mg/day or less. In this study, literature databases were searched through June 2019. Studies that reported the incidence or prevalence of side effects of amiodarone were included. Effect estimates from individual studies were extracted and combined using the random-effects generic inverse variance method of DerSimonian and Laird. A total of 10 observational cohort studies involving 901 patients were included in the analysis. The pooled estimated incidence of overall side effects for low-dose amiodarone was 0.17 [95% confidence interval (CI): 0.12–0.22]. In addition, the pooled estimated incidence of side effects requiring medication discontinuation was 0.06 (95% CI: 0.03–0.11). As compared with 200 mg/day of amiodarone, the pooled estimated incidence of overall side effects was 0.11 (95% CI: 0.04–0.27), while the incidence of side effects requiring medication discontinuation was 0.02 (95% CI: 0.01–0.06) for the dose of 100 mg/day. In conclusion, very-low-dose amiodarone displays a low incidence of significant side effects requiring medication discontinuation.

## Introduction

Amiodarone is commonly utilized for treating both supraventricular and ventricular arrhythmias. While this drug is a very effective antiarrhythmic agent, it also leads to many well-known side effects involving a variety of organs such as the thyroid, liver, lungs, and eyes including many that are dose- and duration-dependent.^1^ Therefore, the use of amiodarone must be balanced between the drug’s potentially serious adverse effects and its antiarrhythmic effects. However, current guidelines still recommend that amiodarone be chosen as the first-line therapy in some patient groups.^2^

Some known adverse effects of amiodarone may be related to the dose and duration. In 1997, a meta-analysis of low-dose amiodarone, defined as less than 400 mg/day, reported a higher rate of drug discontinuation as compared to placebo (22.9% versus 15.4%).^3^ With the development of new therapeutic agents, catheter ablation, and implantable cardioverter-defibrillators (ICDs) for the management of arrhythmias, a lower dose of amiodarone is often sufficient, and studies have shown a degree of clinical efficacy in using a low dose to suppress arrhythmias.^4,5^ A survey in Europe also suggested that very-low-dose amiodarone, defined as 100 mg/day or less, is commonly used to treat arrhythmia.^6^ However, there are no well-designed randomized studies examining the efficacy and safety of very-low-dose amiodarone. Still, many observational studies show promising findings in terms of the safety of very-low-dose amiodarone.^4,7^

Given the above, we conducted the present systematic review and meta-analysis to assess the incidence, prevalence, and odds of side effects of low-dose (≤ 200 mg/day) and very-low-dose (≤ 100 mg/day) amiodarone treatment regardless of arrhythmia indication.

## Methods

### Literature review and search strategy

The protocol for this meta-analysis was registered with the International Prospective Register of Systematic Reviews (no. CRD42018089481). A systematic literature search of MEDLINE (1946 to March 2019), EMBASE (1988 to March 2019), and the Cochrane Database of Systematic Reviews (database inception to March 2019) was conducted to identify studies evaluating associations of amiodarone and side effects in patient with all types of arrhythmias. The systematic literature review was undertaken independently by two investigators (R. C. and R. K. T.), applying a search approach that incorporated variations of “amiodarone” or “side effect” and “safety,” which are covered in **Table 1**. No language limitation was applied. A manual search for conceivably relevant studies among the references of the included articles was also performed. This study was conducted according to the Preferred Reporting Items for Systematic Reviews and Meta-Analysis statement.

### Selection criteria

Eligible studies included cross-sectional, case–control, or cohort studies that assessed the associations of side effect and safety and provided the effect estimates of incidence, prevalence, odds ratios (OR), relative risks (RR), or hazard ratios (HR) with 95% confidence intervals (CIs). Study size did not dictate inclusion status. Retrieved articles were individually reviewed for their eligibility by the two investigators noted previously. Discrepancies were discussed and resolved by mutual consensus. The Newcastle–Ottawa quality assessment scale was used to appraise the quality of case–control studies and the outcomes of interest in cohort studies.

### Data abstraction

A structured data collection form was adopted to compile information from each study including title, year of the study, name of the first author, publication year, country where the study was conducted, demographic and characteristic data of study subjects, exposure measurement, methods used to identify atrial fibrillation, definitions of side effects, and diagnostic methods.

### Statistical analysis

Analyses were performed using the R version 3.5.3 for Mac OS X software program (The R Foundation for Statistical Computing, Vienna, Austria) and the Comprehensive Meta-analysis version 3.3 software program (Biostat Inc., Englewood, NJ, USA). Adjusted point estimates from each study were consolidated by the generic inverse variance approach of DerSimonian and Laird, which determined the weight of each study according to its variance. Given the likelihood of increased interobservation variance, a random-effects model was adopted to assess the pooled prevalence with 95% CIs for the incidence rates of significant side effects caused by low-dose amiodarone treatment. Cochran’s Q test and the I^2^ statistic were applied to determine the between-study heterogeneity. An I^2^ value of 0% to 25% represents insignificant heterogeneity, that of 26% to 50% represents low heterogeneity, that 51% of 75% represents moderate heterogeneity, and that of greater than 75% represents high heterogeneity. The presence of publication bias was evaluated via the Egger test.

## Results

**Figure 1:**
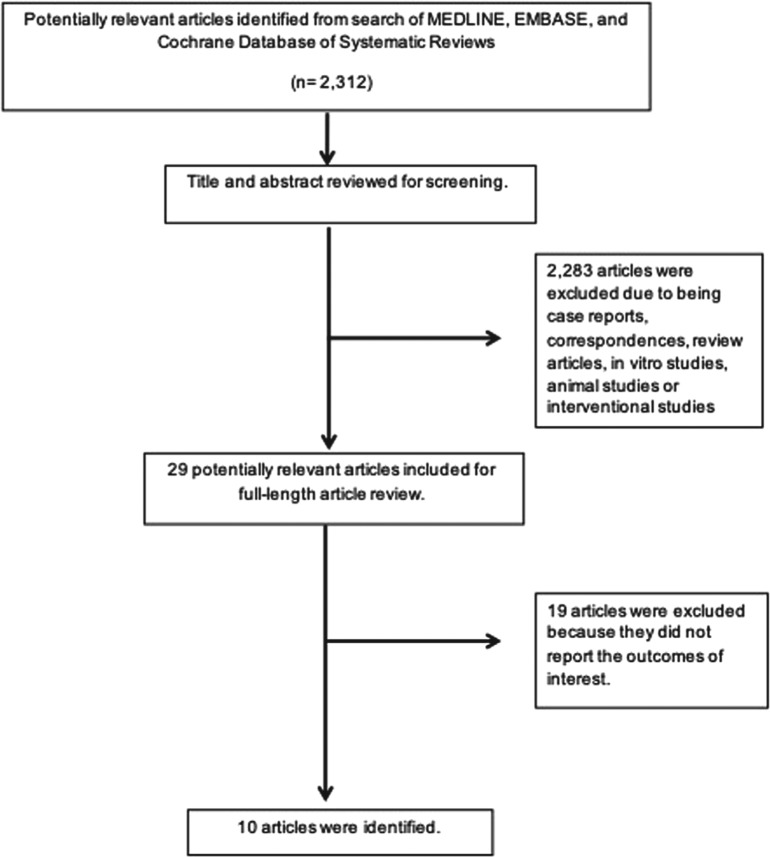
Outline of the search methodology.

A total of 2,312 potentially eligible articles were identified using our search strategy. After the exclusion of 2,283 articles, which included case reports, correspondences, review articles, in vitro studies, animal studies, and interventional studies, 29 articles were left for further full-length review. Nineteen studies did not report outcomes of interest and were excluded. Thus, the final analysis included 10 observational studies—specifically, seven retrospective cohort studies^7–13^ and three prospective cohort studies^4,14,15^—involving a total of 901 patients. The literature retrieval, review, and selection processes are shown in **Figure 1**. The characteristics and quality assessment of the included studies are presented in **Table 2**.

### Definition of amiodarone and its side effects

We arbitrarily defined low-dose amiodarone as an average maintenance dose of 200 mg/day or less. Significant side effects of amiodarone include those that require medication discontinuation due to the failure of conservative treatment of primary organ involvement; hepatotoxicity manifested by a clinically significant transaminitis; significant thyroid disorder diagnosed by laboratory testing along with the clinical manifestation of hyperthyroidism or hypothyroidism^16^; and pulmonary toxicity confirmed by clinical presentation and decreased DLCO without acute heart failure or fibrosis seen on high-resolution pulmonary computed tomography.^2^

Most of the studies (7/10) defined amiodarone-related side effects based on the investigation of clinical presentation and laboratory results.^4,7–9,11,13,15^ Some (3/10) reported side effects without providing diagnostic criteria.^10,12,14^ As such, most of the included studies used standard diagnostic tools for establishing the diagnosis of amiodarone-related side effects.

### Incidence of significant side effects in patients with amiodarone treatment

**Figure 2:**
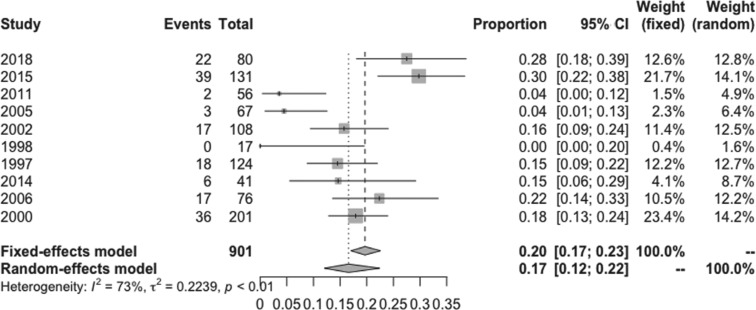
Forest plots of the included studies assessing the incidence of overall side effects of low-dose amiodarone.

**Figure 3:**
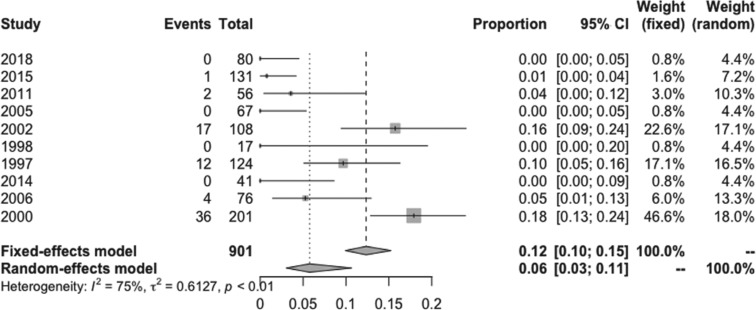
Forest plots of the included studies assessing incidence of serious adverse effects of low-dose amiodarone.

**Figure 4:**
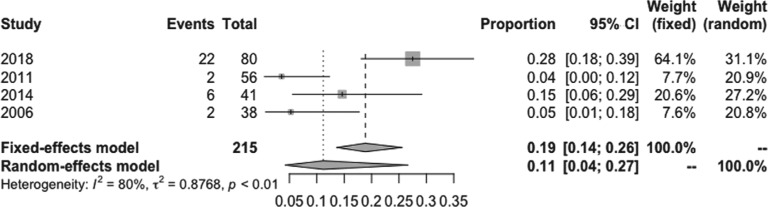
Forest plots of the included studies assessing the incidence of overall side effects of very-low-dose amiodarone.

**Figure 5:**
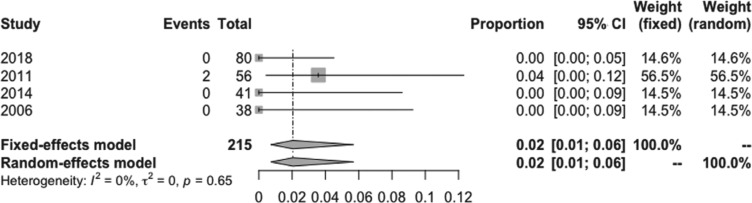
Forest plots of the included studies assessing the incidence of serious adverse effects of very-low-dose amiodarone.

The pooled estimated incidence of overall side effects associated with low-dose amiodarone among the 10 studies included was 17% (95% CI: 12%–22%; I^2^ = 73%) **(Figure 2)**. However, the pooled estimated incidence of a side effect requiring medication discontinuation was 6% (95% CI: 3%–11%; I^2^ = 74.9%) **(Figure 3)**. Compared to 200 mg/day of amiodarone, the pooled estimated incidence of overall side effects with a dose of 100 mg/day of amiodarone was 11% (95% CI: 4%–27%; I^2^ = 80%) **(Figure 4)**, while the pooled estimated incidence of side effects requiring medication discontinuation was 2% (95% CI: 1%–6%; I^2^ = 0%) **(Figure 5)**.^4,15^ None of the included studies reported any instances of amiodarone-related mortality.

### Evaluation for publication bias

Egger’s regression asymmetry test was performed and indicated a presence of publication bias with p < 0.001 for the incidence of significant side effects associated with low-dose amiodarone treatment.

## Discussion

Our study demonstrates a lower incidence of side effects from low-dose amiodarone. The availability of catheter ablation, ICDs, and other AADs have relegated amiodarone to positioning as a second-line therapy and, when it is used, a lower dosage is dispensed as compared with that used in the past, whereas the therapeutic effects are preserved. To our knowledge, our study is the first meta-analysis to investigate the side effects of “low”-dose amiodarone commonly used in current clinical practice. The incidence of side effects requiring medication discontinuation is estimated to be 4%, which is just one-quarter of that reported by Vorperian et al.^3^ Moreover, none of the surveyed studies demonstrated mortality and many only rarely reported irreversible side effects due to amiodarone administration.

Amiodarone is a well-known medication widely used to treat both supraventricular and ventricular arrhythmias.^17^ Moreover, it is the most efficient drug available for maintaining sinus rhythm in patients with atrial fibrillation.^18^ However, its usage is sometimes limited or even entirely prohibited by side effects, including but not limited to thyroid dysfunction, hepatotoxicity, neurotoxicity, and pulmonary toxicity. Based on a pharmacokinetics study, 100 mg/day of amiodarone can similarly suppress nonsustained ventricular tachycardia as compared with the lowest effective dose of 50 mg/day.^19^ Therefore, the lowest effective dose scientifically confirmed by prior research can still provide a therapeutic effect with minimal intolerable side effects.

It is crucial to understand amiodarone’s pharmacokinetics to be able to scrutinize its side effects and toxicity. Due to slow and incomplete gastrointestinal absorption, amiodarone bioavailability varies significantly among different individuals following oral administration.^20^ The amiodarone metabolism by cytochrome P450 3A4 (CYP3A4) in the intestinal wall and gastrointestinal excretion mediated by P-glycoprotein might be major contributors to the overall poor oral bioavailability.^21^ Amiodarone has a high binding affinity to plasma proteins; however, the free fraction is not dependent on either total drug concentration or albumin level.^22^ The exceptionally large volume of distribution of amiodarone is attributable to its high hydrophobicity.^23^ Despite its high fat solubility, the amiodarone plasma concentration might fall by 25% after a few days of drug cessation even after a steady state is achieved.^24^ Likewise, an animal study indicates that obesity might influence the biodistribution and metabolism of amiodarone.^25^ Amiodarone is mainly metabolized by CYP3A4 to desethylamiodarone and subsequent metabolites, then undergoes biliary excretion, with less than 1% being excreted in the urine in an unchanged form.^26^

The dosage and duration of amiodarone are the most important factors influencing the risk of developing side effects. Prior to the era of amiodarone-related lethal side effects, the appropriate maintenance dosing to treat significant arrhythmias ranged between 200 mg/day and 800 mg/day for months to years.^3,16,27–39^ After studies showed that side effects were dosing-related, lower dosing, which is 100 mg/day to 200 mg/day, was adopted, thus achieving a better balance between the acceptable side effects and satisfactory treatment outcomes.^4,14^ The lowest dosing of amiodarone was reported by Jong et al*.* in 2006 in a study designed to investigate the efficacy of low-dose amiodarone on sinus rhythm maintenance after atrial fibrillation cardioversion.^4^ It is noteworthy that no patients in the lower-dose group had significant side effects requiring drug discontinuation. Hence, these safety data encourage the usage of very-low-dose amiodarone.

Regarding side effects, the lack of widely accepted diagnostic criteria and symptom definitions makes it challenging to report the actual incidence of these side effects. Ranging from subclinical to overt clinical manifestations, most of the reported side effects are incidentally revealed through routine laboratory screenings.^40^ Some life-threatening side effects have been reported in patients only on short-term amiodarone, although these extreme adverse events are very rare.^41^ Furthermore, many well-designed studies show that amiodarone is safe under vigilant surveillance. Systematic screening and regular follow-up are the essential elements of these studies. For example, optical coherence tomography is used to detect an early change in retinal fiber layers, which could be reversed by the discontinuation of amiodarone.^33^ Meanwhile, screening and surveillance pulmonary function testing conducted in some studies failed to achieve any benefit in avoiding pulmonary toxicities.^36^ As a result, the available data to clarify the side effects and propose preventive strategies remain under discussion. Moreover, most side effects are diagnosed only after the exclusion of other causes, which could lead to an altered estimation of their incidence rates.

In the last decade, new technologies have been introduced to treat patients with heart diseases who present with concomitant arrhythmias, leading to dramatically decreased morbidity and mortality rates. These new modalities consist of ICDs, cardiac resynchronization therapy systems, and neurohormonal inhibitor medications, which also help to prevent arrhythmias by themselves.^42^ Strategies like these that remain in continued development could further lower the effective dose of amiodarone to control arrhythmia-related problems. Lower doses and improved surveillance would, in theory, result in fewer side effects and improvements in quality of life.

Amiodarone remains the most widely used AAD worldwide. In developing countries, amiodarone is primarily used for atrial fibrillation rate control, whereas, in many developed countries, this drug as a rhythm-control strategy is the mainstream treatment option for qualified patients. The major rate-control drugs are digoxin and amiodarone, which account for 80% of the medication regimens prescribed by physicians from developing countries. These reflect the popularity of amiodarone, which has been used commonly in many parts of the world.^35^ Blackman et al. previously conducted a survey in European countries about amiodarone use among physicians and found that 100 mg/day of amiodarone is widely used by cardiologists in the United Kingdom who believe that “very-low-dose amiodarone” could effectively maintain sinus rhythm in patients with paroxysmal atrial fibrillation.^6^

There are some limitations in our study. First, this study was a meta-analysis of observational studies; hence, we cannot draw a conclusion regarding a causal relationship from the results. Also, the common inherent biases in this study design are recall bias and observational bias. Second, the caveat of data interpretation is the heterogeneity of establishing a side effect diagnosis based on different diagnostic tools and criteria. However, we at least demonstrated that actual serious side effects requiring medication cessation do not commonly appear.

In conclusion, our study demonstrates the safety of very-low-dose amiodarone, which has been prescribed worldwide. In addition, the incidence of side effects from our study is lower than that of a prior meta-analysis, which might encourage physicians to begin or continue to use this useful medication, albeit with limitations as appropriate. Further research should be conducted to provide stronger evidence regarding the relationship between low-dose amiodarone and related side effects.

## Figures and Tables

**Table 1:** Search Terms Applied in the MEDLINE, EMBASE, and Cochrane Databases

1. exp amiodarone/
2. amiodarone$.mp
3. 1 or 2
4. exp side effect/
5. adverse$.mp
6. safety.mp
7. 4 or 5 or 6
8. 3 and 7

**Table 2:** Main Characteristics of the Studies Included in the Meta-analysis of Side Effects of Low-dose Amiodarone

	Iwasawa et al.	Takeuchi et al.	McGrew et al.	Kosior et al.	Shiga et al.	Yamada et al.	Lee et al.	Gao et al.	Jong et al.	Roy et al.
Country	Japan	Japan	Germany	Poland	Japan	Japan	Hong Kong	China	China	Canada
Study design	Retrospective	Retrospective	Retrospective	Retrospective	Retrospective	Retrospective (rapid communication)	Retrospective	Prospective randomized	Prospective randomized	Prospective randomized
Year	2018	2015	2011	2005	2002	1998	1997	2014	2006	2000
Total number	80	131	56	67	108	17	124 (VA 36%)	41	76	201
Mean age ± SD	33 years	28 years	N/A	61 ± 11 years	55 ± 13 years	N/A	N/A	33 ± 6 years	66 ± 10 years	65 ± 11 years
Male sex	56%	N/A	N/A	N/A	85%	N/A	N/A	N/A	53%	55%
Exposure definition	90 mg (SVT); 80 mg (VT)	150 mg	< 100 mg	179 ± 42 mg	50–200 (140) mg	191 ± 52 mg	194 ± 48 mg	100 mg (AMD + metoprolol versus metoprolol)	100 mg vs. 200 mg	AMD 186 ± 48 mg vs. sotalol or propafenone
Exposure duration	35 months (SVT); 38 months (VT)	44 months	21 months	12 months	36 months	38 months	32 months	3 months	67 ± 8 months	16 months
Outcome(s)	Thyrotoxicosis (4 patients, 5%); no interstitial pneumonia	30% developed AITD (18% AIT and 12% AIH); 1 patient suddenly died during the acute phase of AIT	2/49 (4%) patients had dyspepsia and needed to discontinue the medication	3/67 (1 for apparent hyperthyroidism and 2 for decreased TSH level)	17/108 (16%) SE required discontinuation of AMD: 10 decrease in DLCO, 3 PF, 1 hyperthyrodism, 2 sinus bradycardia, and 1 MMVT: no life-threatening events were seen	No life-threatening events were seen	12 patients (drug withdrawal, 4 patients had overt hyperthyroid); cumulative incidence of AMD-related SE was 5.8 per 1,000 patient-years; 1 patient presented nonfatal pulmonary fibrosis	6/41 in treatment group (4 patients N/V and 2 for tolerant sinus bradycardia); no need to withdraw medication	15/38 (200 mg), 2/38 (100 mg); no life-threatening or irreversible cases	36/201 (18%) discontinued in amiodarone group (8 GI, 2 CNS, 6 fatigue/insomnia, 2 visual/skin, 1 pulmonary toxicity, 3 thyroid dysfunction) vs. 23/201 (11%) discontinued in sotalol or propafenone group
Method of outcome ascertainment	Scheduled clinical and laboratory investigation (TSH, CXR, PFT)	Scheduled clinical and laboratory investigation (TSH)	Scheduled clinical and laboratory investigation (TSH, CXR, PFT)	Scheduled clinical and laboratory investigation (TSH, CXR, PFT)	Scheduled clinical and laboratory investigation (TSH, CXR, PFT)	N/A	Scheduled clinical and laboratory investigation (TSH, CXR, PFT)	N/A	Scheduled clinical and laboratory investigation (TSH, CXR, PFT)	Scheduled clinical and laboratory investigation (TSH, CXR, PFT)
Quality assessment (Newcastle–Ottawa scale)	S3, C0, O3	S3, C0, O3	S3, C0, O2	S3, C0, O3	S3, C0, O3	S3, C0, O2	S3, C0, O3	15.20 (95% CI: 0.80–279.30)	0.09 (95% CI: 0.02–0.41)	1.70 (95% CI: 0.97–2.99)

AITD: amiodarone-induced thyroid dysfunction; AIT: amiodarone-induced thyrotoxicosis; AIH: amiodarone-induced hypothyroid; AMD: amiodarone; CI: confidence interval; CNS: central nervous system; CXR: chest X-ray; DLCO: diffusing capacity of the lungs for carbon monoxide; GI: gastrointestinal; MMVT: monomorphic ventricular tachycardia; PF: pulmonary fibrosis; PFT: pulmonary function test; S, C, O: selection, comparability, and outcome; SD: standard deviation; SE: side effect; SVT: supraventricular tachycardia; TSH: thyroid-stimulating hormone; VA: ventricular arrhythmia; VT: ventricular tachycardia.
